# Confirmatory factor analysis of adverse childhood experiences (ACEs) among a community-based sample of parents and adolescents

**DOI:** 10.1186/s12887-020-02063-3

**Published:** 2020-04-21

**Authors:** Tracie O. Afifi, Samantha Salmon, Isabel Garcés, Shannon Struck, Janique Fortier, Tamara Taillieu, Ashley Stewart-Tufescu, Gordon J. G. Asmundson, Jitender Sareen, Harriet L. MacMillan

**Affiliations:** 1 Departments of Community Health Sciences and Psychiatry, University of Manitoba, S113-750 Bannatyne Avenue, Winnipeg, Manitoba R3E 0W5 Canada; 2 Department of Community Health Sciences, University of Manitoba, Winnipeg, Canada; 3 Department of Community Health Sciences, University of Manitoba, Winnipeg, Canada; 4 Department of Community Health Sciences, University of Manitoba, Winnipeg, Canada; 5 Department of Community Health Sciences, University of Manitoba, Winnipeg, Canada; 6 Department of Community Health Sciences, University of Manitoba, Winnipeg, Canada; 7 Departments of Community Health Sciences and Psychiatry, University of Manitoba, Winnipeg, Canada; 8 Department of Psychology and Anxiety and Illness Behaviours Lab, University of Regina, Regina, Canada; 9 Department of Psychiatry, University of Manitoba, Winnipeg, Manitoba Canada; 10 Department of Psychiatry and Behavioural Neurosciences, and of Pediatrics, Offord Centre for Child Studies, McMaster University, Hamilton, Ontario Canada

**Keywords:** Child maltreatment, Physical abuse, Sexual abuse, Neglect, Spanking, Exposure to intimate partner violence, ACEs, Household challenges

## Abstract

**Background:**

Despite increased understanding of Adverse Childhood Experiences (ACEs), very little advancement has been made in how ACEs are defined and conceptualized. The current objectives were to determine: 1) how well a theoretically-derived ACEs model fit the data, and 2) the association of all ACEs and the ACEs factors with poor self-rated mental and physical health.

**Methods:**

Data were obtained from the Well-Being and Experiences Study, survey data of adolescents aged 14 to 17 years (*n* = 1002) and their parents (*n* = 1000) in Manitoba, Canada collected from 2017 to 2018. Statistical methods included confirmatory factor analysis (CFA) and logistic regression models.

**Results:**

The study findings indicated a two-factor solution for both the adolescent and parent sample as follows: a) child maltreatment and peer victimization and b) household challenges factors, provided the best fit to the data. All original and expanded ACEs loaded on one of these two factors and all individual ACEs were associated with either poor self-rated mental health, physical health or both in unadjusted models and with the majority of findings remaining statistically significant in adjusted models (Adjusted Odds Ratios ranged from 1.16–3.25 among parents and 1.12–8.02 among adolescents). Additionally, both factors were associated with poor mental and physical health.

**Conclusions:**

Findings confirm a two-factor structure (i.e., 1) child maltreatment and peer victimization and 2) household challenges) and indicate that the ACEs list should include original ACEs (i.e., physical abuse, sexual abuse, emotional abuse, emotional neglect, physical neglect, exposure to intimate partner violence (IPV), household substance use, household mental health problems, parental separation or divorce, parental problems with police) and expanded ACEs (i.e., spanking, peer victimization, household gambling problems, foster care placement or child protective organization (CPO) contact, poverty, and neighborhood safety).

## Background

Adverse childhood experiences (ACEs) typically describe adversity that has occurred in childhood and often includes child maltreatment and other household challenges. In 1998, Felitti and colleagues published the first research article using Wave I data from the original ACEs Study, which included eight ACEs: emotional abuse, physical abuse, sexual abuse, household member with substance use problems, household member with mental illness, mother treated violently, household criminal behavior, and parental separation or divorce [1, 2]. In Wave II of the original ACEs Study, the number of ACEs was expanded to 10 with the addition of physical neglect and emotional neglect [3]. Since the 1998 ACEs Study publication, the literature on ACEs has grown substantially. What is currently known is that ACEs are common [1–3] and are associated with poor mental health conditions, [4–6] physical health conditions, [1, 3, 7–11] and at-risk behaviours [12–14]. The original ACEs Study served as the foundation for growth of a large body of research furthering our understanding of the association between childhood adversity and health and behavioral outcomes. However, limitations of the original ACEs Study have also been noted, including an unrepresentative sample and a narrow definition of childhood adversity [15]. Importantly, there has been no theoretical or empirical evidence published to indicate why 10 specific experiences were chosen as ACEs in the original ACEs Study data collection. However, these 10 ACEs have been theoretically group together and typically conceptualized into two constructs: 1) child maltreatment ACEs, including physical abuse, sexual abuse, emotional abuse, emotional neglect, and physical neglect, and 2) household challenges or dysfunctions, including parental divorce, mother treated violently or exposure to intimate partner violence (IPV), household member with substance use problems, household member with mental health problems, and household member incarceration [1, 2].

Despite increased understanding of ACEs, very little advancement has been made over the past two decades with regards to how ACEs are defined and conceptualized. Interestingly, many of the studies in this area continue to use the original list of 10 ACEs [16–20]. Other studies only include some of the original 10 ACEs, usually a function of what data can be collected or what constructs are available in existing datasets [13, 21–26]. This means that some studies will examine fewer than the 10 original ACEs. In addition, other studies will examine some or all of the original ACEs as well as including additional adverse experiences that one may experience during childhood. The original list of ACEs, as well as all of these variations of what are considered ACEs, have emerged over the past two decades with no or very little empirical rigor to inform how ACEs are conceptualized.

More recently, Finkelhor and colleagues have conducted studies in an effort to advance knowledge in this area [27, 28]. For example, Finkelhor and colleagues conducted research with the aim of generating a more comprehensive ACEs list by examining significant relationships between the original ACEs items and additional adverse experiences with distress [27, 28] and overall self-perceived physical health [28]. The findings indicated that additional adverse experiences explained more variance in the nested models and the consistent findings across both studies suggested adding neighborhood danger or community violence, poverty, peer victimization, and peer social isolation to the ACEs list. Although these adjusted models did indicate that ACEs in the revised ACEs list were associated with distress and poor physical health outcomes, what was not assessed in this study was the factor structure of these ACEs and how well the individual ACEs empirically cluster together to represent the underlying constructs.

Other researchers have also suggested expanding the ACEs, albeit without empirical evidence derived from factor analyses. The list of expanded ACEs includes witnessing community violence or living in an unsafe neighborhood, [29] parental death, [30] major childhood illness, [30] dating violence, [30] discrimination, [29, 31] unsafe neighborhoods, [29, 31] peer victimization, [29, 31], and placement in foster care [29–31]. Furthermore, it has been recommended that parental divorce or separation should no longer be considered amongst the ACEs since it is not currently an unusual event in society [27]. Therefore, an examination of the factor structure may indicate additional variables that load together and might reveal the possibility of some ACEs not loading on a factor.

To date, only a few studies have been conducted to advance knowledge of defining ACEs using factor analysis. In 2014, Ford and colleagues used ACEs surveillance data from the United States of America to examine the factor structure using exploratory factor analysis and then confirmatory factor analysis of eight of the original ACEs (excluding physical neglect and emotional neglect) and concluded that a three-factor solution existed: 1) household dysfunction (i.e., household member substance use problems, household member mental health problems, household member incarceration, and parental separation or divorce), 2) emotional/physical abuse, and 3) sexual abuse [32]. In 2017, Afifi and colleagues extended this work using the original ACEs Study data and a confirmatory factor analysis and found that spanking also loaded on the emotional/physical abuse factor and accounted for additional variance in the association with drug use, moderate to heavy drinking, and suicide attempts [33]. The conclusion from this work was that spanking should be included in the ACEs list in efforts for violence prevention.

Another study with a sample of low-income women from Wisconsin used exploratory factor analysis to determine how the 10 original ACEs and seven additional ACEs empirically grouped together [34]. These data indicated a two-factor solution when examining the original 10 ACEs consistent with the theoretical child maltreatment and household challenges constructs. When including the additional ACEs, a four-factor structure supporting the original 10 ACEs plus six additional ACEs as follows: 1) physical abuse, sexual abuse, emotional abuse, domestic violence, peer victimization, violent crime, household substance use, and household mental health problem; 2) emotional neglect and physical neglect; 3) financial problems, food insecurity, and homelessness; and 4) household incarceration, parental absence, and parental separation or divorce. This factor structure is not consistent with previous work, which may be due to the unrepresentative nature of the sample of low-income women as well as using eigenvalues lower than 1, which may overestimate the number of factors [35]. Finally, another study was conducted with a focus on differences among siblings in a sample of older adults (mean age = 59 years) and found evidence of different factor structures for within-family and between-family models [36]. As well, the samples used to generate the findings from these two latter studies were very specific (i.e., low income women and older sibling sample) and may have limited capacity to advance knowledge. More research in this area is needed using high quality community and population-based samples.

To further advance knowledge, parental problem gambling may be a potential childhood adversity that warrants further consideration. Problem gambling refers to gambling behaviour that has a negative impact on the gambler, other people in his or her social network, or the community [37]. Previous research has found problem gambling to be associated with dysfunction in family relationships, [38] family violence, [39, 40] and unsafe or unstable family environments [41]. Problem gambling is also associated with mental health conditions and substance use problems [39, 42–44]. Considering the above associations, parental gambling problems may also be an important addition to the ACEs list that should be studied empirically.

Although the original ACEs Study has been criticized for using a narrow definition of ACEs, [15] current research should not simply focus on developing a long and exhaustive list of ACEs. Such a list would be impractical for research and practice. Instead, one way to advance the field would be to look at the empirical structure of the original 10 ACEs along with other possible adverse experiences that are selected thoughtfully and based on theoretical perspectives and findings from previous studies. This would require preforming a confirmatory factor analysis rather than an exploratory factor analysis to test theorized structure and relations between the latent variables that underlie the data [45]. As well, it is important to recognize the difference between developing an ACEs tool and furthering our understanding of how ACEs should be conceptualized. The latter is the focus of the present study. More specifically, we are not validating the original 10 item ACEs tool with this work. Rather, we are addressing an important gap about what should be considered an ACE to inform the expansion of the list using empirical evidence.

When reviewing the current literature on expanding the original list of ACEs there are several adverse experiences that are consistently mentioned and also those with the best evidence for expanding the list. The additional ACEs selected for further consideration were based on an Ecological Systems Theory (described below) and the current literature, they included: poverty, [27–29, 31, 34] peer victimization, [27–29, 31, 34] foster care or contact with child protective organization, [29–31] neighborhood violence, [27, 28] and spanking [33, 46]. In addition, similar to household mental health problems and the links between gambling problems and household violence, [40, 47] it is important to also consider parental problem gambling as an ACE.

Various multi-disciplinary theoretical perspectives have been put forth to conceptualize and operationalize ACEs, including attachment theory [48] (developmental systems and developmental resilience life course theories [49]. While these theoretical perspectives may share overlapping characteristics, not all of these perspectives emphasize the influence of both the individual-familial and social-environmental experiences of early life adversity, which together, represent a foundational characteristic of the ACEs research.

One of the most comprehensive theoretical frameworks that continues to guide the conceptualization of the *current* ACEs research is based on Ecological Systems Theory [50]. A framework based on this theoretical perspective examines experiences of early life adversity from the individual, familial- and social-environment contexts embedded within the broader cultural and structural environment and temporal context. Notably, this framework considers individual-familial and social-environmental adverse experiences together, recognizing that these elements are not mutually exclusive regardless of proximity of context (i.e., distal experiences versus proximal experiences). The ecological systems theoretical perspective provides a strong rationale for the need to re-evaluate the relevance of the original ACEs first identified over 20 years ago, and to examine new and expanded ACEs with samples and populations in diverse contexts that differ from the original ACEs cohort collected over 20 years ago.

The objectives of the current study were to use a community sample of parents and adolescents to examine: (1) the fit of a theoretically-derived model of the original ACEs along with potential expanded ACEs selected based on theoretical perspectives and evidence from previous research (i.e., poverty, spanking, contact with child protective organizations (CPO), parental gambling problems, peer victimization, and neighborhood safety), and (2) if individual ACEs (original and possible expanded ACEs) as well as the ACEs factors are associated with poor self-rated mental and physical health for both parents and adolescents separately.

## Methods

### Data and sample

Data were obtained from the Well-Being and Experiences (WE) Study, which involved a baseline survey of adolescents aged 14 to 17 years (*n* = 1002) and their parents (*n* = 1000) in Manitoba, Canada. Two parents did not complete the survey, which is why the adolescent and parent sample sizes differ. Since the adolescent and parent data were not linked, the additional adolescents were included in this analysis. The sampling design for the WE Study used random digit dialing (21%) and convenience sampling (79%) such as referrals, and community advertisements. From the random digit dialing portion of the sample, 83% were interested in participating in the study and 17% refused to participate. Of the 83, 97% were ineligible because an adolescent aged 14 to 17 years old did not live in the household. Of those who were eligible, 63% consented and completed the survey. Differences in the distribution of the data were not found based on sample method for age, grade, ethnicity, emotional abuse, emotional neglect, exposure to verbal IPV, household substance use, household mental illness, parental trouble with police, parental gambling, foster care or child protective organization [CPO], poverty, and neighbourhood safety. The Forward Sortation Area (FSA) from postal codes was used to ensure the sample was closely representative of Winnipeg, Manitoba, the largest city in the province with a population of approximately 753,700 and surrounding rural areas. Data collection was monitored to ensure that the adolescent sample closely approximated the general population with regard to sex (adolescents), household income, and ethnicity, following the Statistics Canada (2017) census profile. As with other studies using similar designs, the person most knowledgeable of the adolescent was asked to complete the survey [51]. In the majority of cases, the person most knowledgeable was the mother. This means that our adult sample is mostly women and, therefore, the parent sample is not representative of the general population. Data were collected between July 2017 and October 2018. Parents and adolescents self-completed a questionnaire at a research facility in private separate rooms. Parents did not have access to adolescent responses. All respondents provided informed consent to participate and were aware that they could withdraw from the study at any time. Parents and adolescents were compensated $50 and $30, respectively for their time and travel expenses. Ethical approval was provided from the Health Research Ethics Board at the University of Manitoba.

### Measurements

#### Adverse childhood experiences (ACEs)

*Original ACEs.* For parents, all 10 original ACEs (i.e., physical abuse, sexual abuse, emotional abuse, physical neglect, emotional neglect, exposure to IPV, household substance abuse, household mental illness, parental separation or divorce, and parental trouble with police) that they experienced in their own childhood were assessed in the sample. However, not all constructs were measured using the ACEs checklist. Rather, more detailed assessments of these experiences were used when available. Childhood physical abuse, sexual abuse, emotional abuse, physical neglect, and emotional neglect were measured using the Childhood Trauma Questionnaire, [52] which included five items for each of the following: physical abuse, sexual abuse, emotional abuse, physical neglect, and emotional neglect. These items asked about the parents’ experiences when growing up and were dichotomized as recommended by the guidelines for classification of the CTQ scale total scores [52]. Exposure to physical IPV was assessed using an adapted item from the Childhood Experiences of Violence Questionnaire (CEVQ) asking the respondents if, before age 16 years, they heard a parent, step-parent or guardian hit each other or another adult in their home [53]. The remaining four ACEs were from the ACEs Study or adapted from the ACEs Study [1]. More specifically, household substance abuse was assessed with two items asking if, before age 16, a parent or other adult living in their home ever had problems with 1) alcohol or spent a lot of time drinking or being hung over and 2) drugs. Household mental illness was assessed by asking if, before age 16, a parent or other adult living in their home ever had mental health problems like depression or anxiety. Parental separation or divorce was assessed by asking if their biological parents were ever separated or divorced before the respondent was 16 years old. Finally, rather than asking about parental incarceration, respondents were asked if, before age 16 years, a parent or other adult living in their home ever had problems with the police.

For adolescents, seven of the original ACEs were asked, excluding physical abuse, sexual abuse, and physical neglect due to the reporting laws for this age group since the WE Study data were not anonymously collected. Emotional neglect was measured using five items from the CTQ subscale and modified to the present tense [52]. Emotional abuse was assessed with one item asking how many times in the past 12 months a parent or other adult living in their home said hurtful or mean things to the respondent. Emotional abuse was dichotomized as once a month or more often versus several times a year or less often. Exposure to verbal IPV was assessed using one item from the CEVQ asking how often in the past 12 months the respondent has ever seen or heard adults say hurtful or mean things to another adult in their home [53]. Exposure to verbal IPV was also dichotomized as once a month or more often versus several times a year or less often. The remaining original ACEs (i.e., household substance use, household mental disorders, parental separation or divorce, and parental trouble with police) were all assessed with the same items used in the adult sample indicated above.

Potential Expanded ACEs.

*Spanking.* Parents and adolescents were asked how often they remember being spanked by an adult (or parent or caregiver) in a typical year when they were 10 years old or younger, using an item adapted from the CEVQ [53]. Spanking was dichotomized as *two* to three times a year or more frequently versus *once a year* or less frequently.

*Parental gambling.* Parents and adolescents were asked whether a parent or other adult living in the home ever had problems with gambling. For parents in the sample, this question referred to when they were younger than 16 years. Gambling was dichotomized as yes versus no.

*Foster Care or Child Protective Organization (CPO) contact.* Both parents and adolescents were asked about contact with a CPO (e.g., social services, child welfare, children’s aid, or the Ministry) due to difficulties in the home (for parents, before they were 16 years). In addition, adolescents in the sample were asked if they had ever been placed in a foster home or group home. Foster care and CPO contact were dichotomized as *yes* versus *no*, and adolescents could indicate yes to one of the items or both.

*Poverty.* Two items were used to assess the frequency of financial difficulty for the participant’s family (before 16 years for the parent respondents and presently for the adolescent respondents). The first item asked specifically about difficulty paying rent or the mortgage on the house and the second item asked about difficulty paying for basic necessities like food or clothing. Each item was dichotomized as *sometimes* or more often as a proxy for poverty versus rarely or never. Participants who indicated frequent financial difficulty for either one or both items were coded as *yes* for this proxy of poverty.

##### Peer victimization

Parents were asked two questions about peer victimization: 1) *Sometimes kids get hassled or picked on by other kids who say hurtful or mean things to them. Before the age of 16, how many times did this happen to you?* and 2) *Sometimes kids get physically pushed around, hit or beaten up by other kids or a group of kids. Before the age of 16, how many times did this happen to you?* Both items were dichotomized, with the first item coded as yes if the participant indicated that this occurred more than 10 times and the second item coded yes if it occurred 3 to 5 times or more. Adolescents were asked about seven forms of peer victimization in the past 12 months, including: 1) bullied, picked on you, or said means things about you, or threatened you through texting or the Internet (e.g., posted something on Facebook or other social media, or sent texts or emails); 2) made fun of you, called you names or insulted you in person or behind your back, but excluding texting, email, social media, or online posting or communications; 3) spread rumors about you in person or behind your back, but excluding texting, email, social media, or online posting or communications; 4) pushed you, shoved you, tripped you, or spit on you; 5) said something bad about your race, culture, or religion in person or behind your back, but excluding texting, email, social media, or online posting or communications; 6) said something bad about your sexual orientation or gender identity in person or behind your back, but excluding texting, email social media, or online posting or communications; and 7) said something bad about your body shape, size, or appearance in person or behind your back, but excluding texting, email, social media, or online posting or communications. Response options were: never, 1 or 2 times a year, 3 to 6 times a year, 7 to 11 times a year, once a month, a couple times a month, once a week, a couple times a week, and every day. A single indicator for peer victimization was coded according to whether the participant reported experiencing any of these items once a month or more often.

##### Neighborhood safety

Neighborhood safety was only assessed among adolescents. Respondents were asked to indicate how much they agree with the following statement: *I feel safe in my community*. If participants indicated that they strongly disagree or disagree with the statement, this item was coded as not safe.

#### Physical and mental health

Two items were used to assess respondents’ self-rated physical health (i.e., *In general, how would you rate your physical health?*) and mental health (i.e., *In general, how would you rate your mental health?*). Response categories were dichotomized as 1) excellent, very good, or good versus 2) fair or poor.

Sociodemographic covariates.

The sociodemographic characteristics of parents and adolescents that were included as covariates in the logistic regression models were sex (male or female), age in years, race/ethnicity (white only, white and another race or ethnicity, and other/multi-race or ethnicity), and household income ($49,999 or less, $50,000 to $99,999, $100,000 to $149,999, and $150,000 or more).

Statistical Analyses.

Confirmatory factor analysis (CFA) was conducted separately for parents and adolescents to examine how the expanded list of potential ACEs (i.e., spanking, parental gambling, foster care or CPO contact, poverty, peer victimization, and neighborhood safety) corresponded with the original ACEs items. Existing theoretical groupings in the ACEs literature identify two ACEs categories, including child maltreatment and peer victimization and household dysfunction or challenges [1, 10]. Based on this theoretical framework and the Ecological Systems theory, a two-factor model was specified for parents and adolescents in a following CFA. Additionally, we tested alternative one-factor and a three-factor models to determine the factor structure with the best fit. Models were standardized using the unit variance identification (UVI) constraint and estimated using mean- and variance-adjusted weighted least squares (WLSMV) estimation. Model fit was assessed with the model chi-square test (X^2^), Root Mean Square Error of Approximation (RMSEA) and its 90% confidence interval (CI), Comparative Fit Index (CFI), Tucker-Lewis Index (TLI), and Standardized Root Mean Square Residual (SRMR). CFA was conducted in Mplus 8.0 [54]. Finally, logistic regression analyses were conducted to examine the associations of each of the individual ACEs (i.e., all original ACEs in addition to spanking, parental gambling, foster care or CPO contact, poverty, peer victimization, and neighborhood safety) and the confirmed factors with self-rated physical and mental health status. The models were first run unadjusted and then adjusting for sociodemographic characteristics. Inter-item tetrachoric correlations of ACEs among parents and adolescents were also computed.

## Results

Table 1 provides the sociodemographic characteristics of the study sample. Among parents, 87% were female with a mean age of 45 years. For adolescent respondents, 52% were female with a mean age of 15.3 years. Among parents and adolescents, 89.1 and 84.8% experienced one or more ACEs, respectively. The Cronbach’s alpha for parents for all 15 ACEs items was .81. The alpha for adolescents for all 13 ACEs items was .71.

**Table 1:** Prevalence of sociodemographic characteristics and original and expanded Adverse Childhood Experiences (ACEs) among parents and adolescents in the sample

Characteristic	Parents (*n* = 1000)	Adolescents (*n* = 1002)
**Sex,**		
Male	13.5	48.3
Female	86.5	51.7
**Age,*mean (SD)***	45.2 (6.0)	15.3 (1.1)
**Ethnicity,**		
White only	68.6	59.1
White and another	10.2	20.7
Other/multi-ethnicity	21.2	20.3
**Household Income**^**a**^**,*%***		
$49,999 or less	20.9	–
$50,000 to $99,999	36.9	–
$100,000 to $149,999	23.3	–
$150,000 or more	18.9	–
**Original ACEs,*%***		
Physical abuse	22.2	NA
Sexual abuse	27.0	NA
Emotional abuse	17.0	22.8
Physical neglect	25.3	NA
Emotional neglect	14.0	7.5
Exposure to IPV	12.6	23.5
Household substance abuse	30.4	16.5
Household mental illness	32.7	37.8
Parental separation or divorce	23.5	27.8
Parental trouble with police	8.6	10.2
**Potential Expanded ACEs,*%***		
Spanking	45.3	30.7
Peer victimization	45.6	24.1
Parental gambling	6.3	3.7
Foster care or CPO contact	7.4	13.9
Poverty	40.0	21.5
Neighbourhood safety	NA	3.6
**Self-Rated Health**		
Fair/Poor Physical Health	16.1%	19.5%
Fair/Poor Mental Health	17.3%	33.2%

^a^Reported by the parent only

**Fig. 1:**
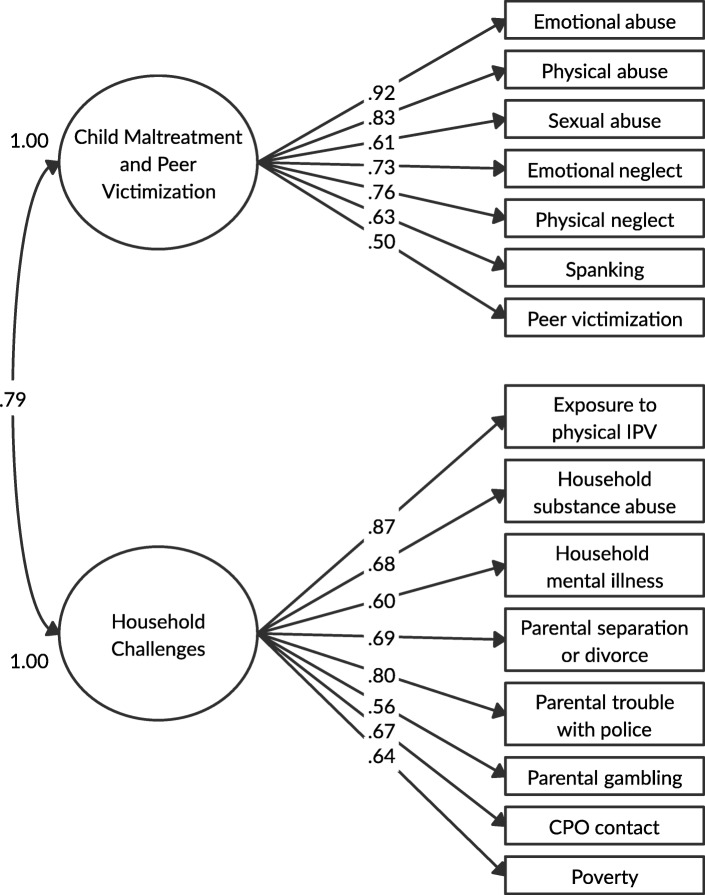
Two-factor CFA model with standardized factor loadings for parent ACEs. *Model fit:* X^2^_(89)_ = 316.8, *p* < .001; RMSEA = .051, 90% CI = .045–.057; CFI = .952; TLI = .943; SRMR = .071. *Abbreviations:* IPV = intimate partner violence; CPO = child protective organization

In the initial CFA, the two-factor model for parents was first specified and was found to have acceptable fit (X^2^ (89) = 341.5; *p* < .001; RMSEA = .053, 90% CI = .047–.059; CFI = .947; TLI = .937; SRMR = .073). The modification index for moving physical IPV to Factor 2 was 39.5 (*p* < .001 based on 1 degree of freedom). However, after initial assessment, the model was re-specified to examine whether exposure to IPV had a better factor loading on factor 2 compared to factor 1 since exposure to IPV has been included in previous work as a form of child maltreatment and a household challenge [46, 55, 56]. Overall, the factor loadings were improved when exposure to physical IPV was moved to factor 2. More specifically, in the first model with exposure to physical IPV on factor 1, the standardized factor loading ranged from 0.497 to 0.903 and for factor 2 ranged from 0.574–0.828. When exposure to physical IPV was moved to factor 2, the standardized factor loading for factor 1 and factor 2 ranged from 0.503 to 0.920 and 0.562–0.871, respectively. As well, when exposure to physical IPV was moved to factor 2 the model fit was acceptable (X^2^ (89) = 316.8; *p* < .001; RMSEA = .051, 90% CI = .045–.057; CFI = .952; TLI = .943; SRMR = .071). We also tested two alternative models (i.e., a one-factor model and a three-factor model) to determine the best fit. The one-factor solution had a poorer fit based on fit statistics (X^2^ (90) = 443.0; *p* < .001; RMSEA = .063, 90% CI = .057–.069; CFI = .926; TLI = .913). The three-factor model found that although the model fit was similar to the two-factor model (X^2^ (87) = 311.6; *p* < .001; RMSEA = .051, 90% CI = .045–.057; CFI = .953; TLI = .943; SRMR = .069), the correlation between factor 2 and factor 3 was high (*r* = .99), suggesting the that the third factor is redundant. Therefore, we chose to retain the two-factor solution for parent ACEs shown in Fig. 1 as the best overall model. Standardized factor loadings for all ACEs items were moderate to strong, ranging from .50 to .92 on factor 1 (child maltreatment and peer victimization) and .56 to .87 on factor 2 (household challenges). Examination of the factor loadings suggests that the expanded ACEs are strongly related to the respective child maltreatment and household challenges constructs, with spanking (λ = .63) and peer victimization (λ = .50) on child maltreatment and parental gambling (λ = .56), CPO contact (λ = .67), and poverty (λ = .64) on household challenges. Based on the how the variables factored, factor 1 is referred to as child maltreatment and peer victimization and factor 2 is referred to as household challenges.

**Fig. 2:**
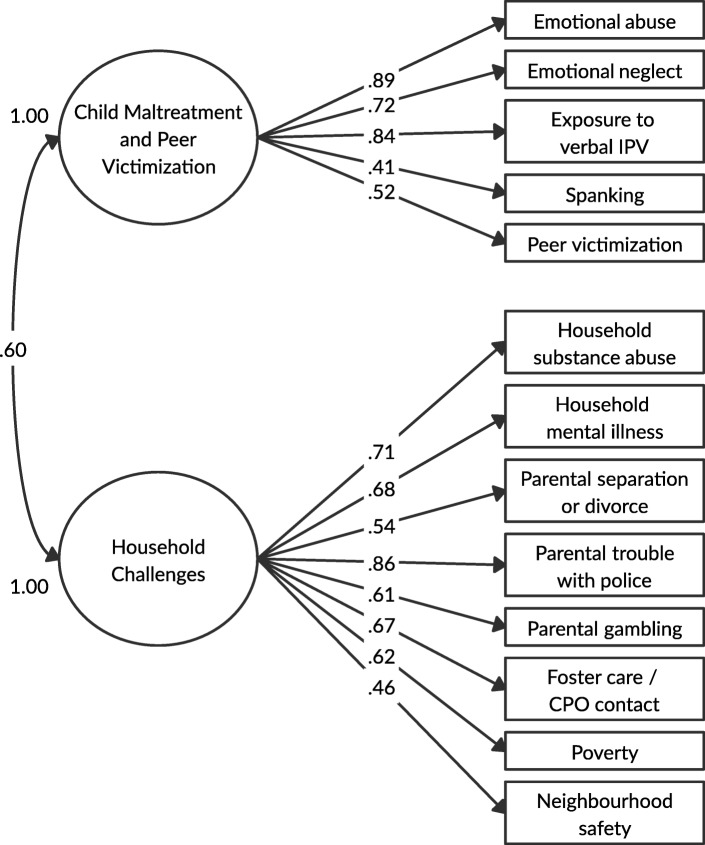
Two-factor CFA model with standardized factor loadings for adolescent ACEs. *Model fit:* X^2^_(64)_ = 144.3, *p* < .001; RMSEA = .035, 90% CI = .028–.043; CFI = .962; TLI = .954; SRMR = .081. *Abbreviations:* IPV = intimate partner violence; CPO = child protective organization

Figure 2 presents the two-factor CFA model of adolescent ACEs. The model was found to have acceptable fit (X^2^ (64) = 144.3; *p* < .001; RMSEA = .035, 90% CI = .028–.043; CFI = .962; TLI = .954; SRMR = .081). We also re-specified the adolescent two-factor model by moving exposure to verbal IPV from factor 1 to factor 2, but found this move did not improve factor loadings and correlation between factors became higher (*r =* .60 versus *r* = .82) when exposure to verbal IPV was on factor 2. As well, having verbal IPV on factor 2 did not find an improvement in the model and fit statistics were overall not adequate (X^2^ (64) = 293.6; *p* < .001; RMSEA = .060, 90% CI = .053–.067; CFI = .892; TLI = .869; SRMR = .101). We then tested the three-factor model, which had acceptable fit (X^2^ (62) = 138.4; *p* < .001; RMSEA = .035, 90% CI = .027–.043; CFI = .964; TLI = .955; SRMR = .078), but factor 3 was found to be highly correlated with factor 2 (*r* = 1.00). We, therefore, retained the more parsimonious two-factor solution as shown in Fig. 2. Standardized factor loadings for all ACEs items were good to strong, ranging from ranged from 0.41 to 0.89 for factor 1 (child maltreatment and peer victimization) and 0.46 to 0.86 for factor 2 (household challenges). The correlation between factor 1 and factor 2 was 0.60. There was acceptable factor interpretability for the expanded ACEs, with spanking (λ = .41) and peer victimization (λ = .52) on the child maltreatment construct, and parental gambling (λ = .61), foster care/CPO contact (λ = .67), poverty (λ = .62), and neighbourhood safety (λ = .46) on the household challenges construct. Similar to the parent models and based on the factor loadings, factor 1 is referred to as child maltreatment and peer victimization and factor 2 is referred to as household challenges.

Table 2 provides the results for the associations between individual ACEs and the two ACEs factors with self-rated mental and physical health among parents and adolescents. Among parents, all individual original and expanded ACEs were associated with an increased likelihood of poor self-rated physical health with the exception of physical neglect. When adjusting for sociodemographic variables, emotional abuse, physical abuse, emotional neglect, exposure to physical IPV, spanking, poverty, and peer victimization remained significantly associated with an increased likelihood of poor self-rated physical health. Among parents, all individual ACEs were associated with increased odds of poor self-rated mental health and all remained significant even after adjusting for sociodemographic variables. Among adolescents, all individual original and expanded ACEs were associated with an increased likelihood of poor self-rated physical health with the exception of spanking. When adjusting for sociodemographic variables, only neighborhood safety was attenuated enough to become non-significant. Among adolescents, all individual ACEs were associated with increased odds of poor self-rated mental health and only spanking became non-significant after adjusting for sociodemographic variables. Associations were also significant between ACEs factors and self-rated physical health and self-rated mental health for parents and adolescents. For parents, child maltreatment and peer victimization was associated with 2.86 (95% CI = 1.62 to 5.05) increased odds of self-rated physical health and 3.19 (95% CI = 1.81 to 5.60) increased odds of self-rated mental health in adjusted models. The factor, household challenges was associated with 1.97 (95% CI = 1.20 to 3.24) increased odds of self-rated physical health and 2.67 (95% CI = 1.61 to 4.44) increased odds of self-rated mental health in adjusted models. For adolescents, child maltreatment and peer victimization was associated with 2.15 (95% CI = 1.41 to 3.26) increased odds of self-rated physical health and 3.00 (95% CI = 2.12 to 4.26) increased odds of self-rated mental health in adjusted models. The factor household challenges was associated with 2.99 (95% CI = 1.81 to 4.95) increase odds of self-rated physical health and 6.09 (95% CI = 3.88 to 9.57) increased odds of self-rated mental health in adjusted models. Table 3 presents the inter-item tetrachoric correlations of ACEs among parents and adolescents.

**Table 2:** Associations of individual Adverse Childhood Experiences (ACEs) with fair or poor self-rated physical and mental health status among parents and adolescents

ACE	Parents				Adolescents			
	Physical Health		Mental Health		Physical Health		Mental Health	
	OR (95% CI)	AOR (95% CI)	OR (95% CI)	AOR (95% CI)	OR (95% CI)	AOR (95% CI)	OR (95% CI)	AOR (95% CI)
***Original ACEs***								
Emotional abuse	3.23*** (2.21, 4.74)	2.66*** (1.76, 4.02)	3.66*** (2.52, 5.33)	3.25*** (2.17, 4.86)	2.26*** (1.57, 3.24)	1.99** (1.35, 2.94)	3.39*** (2.46, 4.68)	2.94*** (2.07, 4.16)
Physical abuse	2.38*** (1.65, 3.42)	2.11*** (1.42, 3.13)	2.34*** (1.64, 3.35)	2.25*** (1.53, 3.30)	NA	NA	NA	NA
Sexual abuse	1.69** (1.18, 2.43)	1.45 ns (0.98, 2.14)	2.58*** (1.82, 3.66)	2.52*** (1.72, 3.67)	NA	NA	NA	NA
Emotional neglect	1.84** (1.19, 2.85)	1.82* (1.15, 2.90)	2.90*** (1.93, 4.35)	3.17*** (2.06, 4.89)	2.80*** (1.69, 4.64)	2.40** (1.38, 4.19)	9.17*** (5.00, 16.82)	8.02*** (4.18, 15.39)
Physical neglect	1.43 ns (0.99, 2.07)	1.16 ns (0.78, 1.74)	2.18*** (1.54, 3.09)	2.14*** (1.47, 3.12)	NA	NA	NA	NA
Exposure to IPV	2.34*** (1.51, 3.61)	1.79* (1.11, 2.88)	2.58*** (1.69, 3.94)	2.24** (1.41, 3.55)	2.05*** (1.43, 2.94)	1.99** (1.35, 2.93)	3.19*** (2.31, 4.41)	2.87*** (2.02, 4.09)
Household substance abuse	1.51* (1.06, 2.15)	1.33 ns (0.91, 1.96)	1.98*** (1.41, 2.78)	1.87** (1.30, 2.70)	1.94** (1.29, 2.91)	1.74* (1.12, 2.71)	2.26*** (1.58, 3.25)	2.02*** (1.37, 2.99)
Household mental illness	1.62* (1.12, 2.36)	1.48 ns (0.99, 2.22)	2.76*** (1.92, 3.96)	2.51*** (1.71, 3.68)	2.36*** (1.64, 3.40)	2.16*** (1.45, 3.21)	5.64*** (4.08, 7.79)	4.79*** (3.37, 6.81)
Parental separation or divorce	1.49* (1.01, 2.20)	1.33 ns (0.87, 2.02)	2.02*** (1.40, 2.93)	1.86** (1.25, 2.76)	1.90*** (1.34, 2.69)	1.88** (1.26, 2.81)	2.15*** (1.58, 2.93)	2.35*** (1.63, 3.38)
Parental trouble with police	2.04** (1.21, 3.42)	1.35 ns (0.75, 2.42)	3.16*** (1.95, 5.12)	2.35** (1.39, 3.99)	2.25** (1.40, 3.62)	1.91* (1.14, 3.19)	3.19*** (2.06, 4.96)	3.41*** (2.10, 5.53)
***Expanded ACEs***								
Spanking	2.15*** (1.52, 3.06)	2.05*** (1.41, 2.97)	2.14*** (1.52, 3.00)	2.10*** (1.46, 3.01)	1.30 ns (0.92, 1.83)	1.12 ns (0.77, 1.63)	1.46* (1.08, 1.96)	1.34 ns (0.97, 1.85)
Parental gambling	1.96* (1.08, 3.57)	1.52 ns (0.81, 2.86)	2.54** (1.44, 4.45)	2.17* (1.20, 3.92)	3.14** (1.55, 6.35)	3.04** (1.46, 6.33)	3.20** (1.59, 6.43)	3.30** (1.55, 7.00)
Foster care or CPO contact^a^	2.48** (1.46, 4.23)	1.50 ns (0.84, 2.70)	3.66*** (2.21, 6.04)	2.50** (1.45, 4.31)	2.57*** (1.72, 3.83)	2.19*** (1.41, 3.40)	2.68***(1.84, 3.90)	2.41*** (1.58, 3.67)
Poverty	2.04*** (1.42, 2.92)	1.64* (1.11, 2.42)	2.14*** (1.51, 3.03)	1.86** (1.28, 2.71)	2.42*** (1.68, 3.51)	1.81** (1.18, 2.79)	2.48*** (1.77, 3.47)	2.57*** (1.71, 3.85)
Peer victimization	2.66*** (1.86, 3.81)	2.67*** (1.83, 3.89)	2.77*** (1.95, 3.93)	2.69*** (1.87, 3.88)	2.01*** (1.41, 2.88)	1.93** (1.31, 2.83)	2.51*** (1.83, 3.44)	2.74*** (1.94, 3.88)
Neighbourhood safety	NA	NA	NA	NA	2.19* (1.08, 4.47)	1.99 ns (0.92, 4.31)	4.13*** (2.03, 8.41)	4.77*** (2.15, 10.57)
***Confirmed Factors***								
Any Child Maltreatment or Peer Victimization^b^	3.04*** (1.77, 5.22)	2.86*** (1.62, 5.05)	3.08*** (1.82, 5.22)	3.19*** (1.81, 5.60)	2.45*** (1.66, 3.62)	2.15*** (1.41, 3.26)	3.14*** (2.27, 4.34)	3.00***(2.12, 4.26)
Any Household Challenge^c^	2.46*** (1.54, 3.94)	1.97** (1.20, 3.24)	3.05*** (1.90, 4.90)	2.67*** (1.61, 4.44)	3.60*** 2.24, 5.79	2.99*** 1.81, 4.95	5.50*** (3.68, 8.22)	6.09*** (3.88, 9.57)

^a^ For parents, CPO contact does not include foster care

^b^ The child maltreatment/peer victimization factor differs for parents and adolescents

^c^ The household challenges factor differs for parents and adolescents

*Abbreviations: IPV* intimate partner violence, *CPO* child protective organization, *OR* odds ratio, *CI* confidence interval, *AOR* odds ratio adjusted for age, sex, ethnicity, and household income (parent-reported household income was included in the adolescent models); NA = not applicable

* < .05; ** < .01; *** < .001; ns = not significant

**Table 3:** Inter-item tetrachoric correlations of Adverse Childhood Experiences (ACEs) among parents and adolescents

	1	2	3	4	5	6	7	8	9	10	11	12	13	14	15	16
1. Emotional abuse	1.00	NA	NA	.64	NA	.81	.25	.29	.16	.32	.38	.30	.32	.32	.46	.31
2. Physical abuse	.70	1.00	NA	NA	NA	NA	NA	NA	NA	NA	NA	NA	NA	NA	NA	NA
3. Sexual abuse	.49	.46	1.00	NA	NA	NA	NA	NA	NA	NA	NA	NA	NA	NA	NA	NA
4. Emotional neglect	.78	.50	.42	1.00	NA	.47	.32	.50	.18	.25	.28	.31	.31	.33	.39	.54
5. Physical neglect	.65	.49	.45	.68	1.00	NA	NA	NA	NA	NA	NA	NA	NA	NA	NA	NA
6. Exposure to IPV	.66	.63	.50	.45	.60	1.00	.42	.35	.17	.42	.22	.32	.24	.42	.32	.19
7. Household substance abuse	.46	.39	.37	.30	.45	.63	1.00	.46	.40	.73	.13	.49	.34	.33	.18	.31
8. Household mental illness	.56	.39	.30	.30	.30	.44	.38	1.00	.42	.43	.23	.37	.45	.46	.29	.30
9. Parental separation or divorce	.42	.44	.41	.25	.42	.56	.48	.44	1.00	.43	.10	.31	.49	.38	.18	.27
10. Parental trouble with police	.49	.40	.41	.29	.54	.64	.69	.51	.60	1.00	.32	.59	.63	.50	.31	.18
11. Spanking	.56	.75	.32	.35	.33	.44	.19	.30	.30	.15	1.00	.13	.30	.19	.20	.17
12. Parental gambling	.39	.30	.22	.32	.33	.42	.49	.34	.37	.56	.16	1.00	.28	.28	.33	.30
13. Foster care or CPO contact	.56	.50	.35	.42	.46	.46	.34	.28	.54	.58	.29	.31	1.00	.43	.22	.14
14. Poverty	.47	.36	.36	.32	.52	.51	.38	.32	.50	.51	.25	.43	.38	1.00	.26	.19
15. Peer victimization	.46	.43	.32	.21	.29	.40	.21	.37	.29	.37	.34	.18	.30	.29	1.00	.35
16. Neighbourhood safety	NA	NA	NA	NA	NA	NA	NA	NA	NA	NA	NA	NA	NA	NA	NA	1.00

*Notes:* Correlations among parents are displayed in the bottom-left section; Correlations among adolescents are displayed in the top-right section; For parents, CPO contact does not include foster care.

*Abbreviations: IPV* intimate partner violence, *CPO* child protective organization, *NA* not applicable

## Discussion

There are several novel findings from the WE Study. First, it is the first study to use both a parent and adolescent sample to assess the empirical factor structure of the original and additional recommended ACEs to inform an updated and evidence-based conceptualization of ACEs. The findings from both parents and adolescents confirm that a two-factor structure provides a good empirical fit to the data that adheres to the original theoretical categorization of ACEs as (a) child maltreatment and peer victimization and (b) household challenges. Second, the current findings support expanding the original ACEs list to include spanking and peer victimization on the child maltreatment and peer victimization factor and parental gambling, CPO contact, poverty, and neighbourhood safety on the household challenges factor. Third, there is no evidence, indicated by low factor loadings, that any of the original ACEs should be removed or that any additional recommended ACEs did not load. Finally, all original and expanded ACEs and each of the ACEs factors were associated with poor self-rated physical and/or mental health.

Throughout the last two decades, the ACEs literature has theoretically categorized 10 ACEs into two groups: child maltreatment and household challenges [1, 10]. Only a small number of studies have empirically examined the factor structure; yet, these studies provide limited opportunity for conclusion or comparison due to the diversity in samples, objectives of the studies, specific methods, and ACEs examined [32, 34, 57]. The current study extends knowledge by providing empirical evidence for the theorized structure and conceptualization of ACEs in both a parent and adolescent sample. What these data indicate is that there is evidence that all original ACEs, including parental separation or divorce, remain relevant and should be considered as ACEs. Notably, due to the low prevalence of parental incarceration, this variable was changed in the current study to parental trouble with police, which may be a less extreme indicator of this type of adversity for a family. Parental trouble with police loaded with other household challenges similar to the theoretical categorization of parental incarceration. Moving forward, it is recommended that parental incarceration be replaced with parental trouble with police.

Previously, spanking has been shown to load with physical/emotional abuse using the original ACEs Study data, which, in part, led to the conclusion that spanking should be considered an ACE [33]. The current study confirms this finding in a parent and adolescent sample. Notably, spanking loaded with higher factor loadings in the parent sample compared to the adolescent sample. This is likely a result of only having an indicator for emotional abuse and not physical abuse in the adolescent sample. Spanking and physical abuse are highly correlated in the parent sample (*r* = .75, see Table 3), which would increase factor loading. Nonetheless, spanking still adequately loaded on the child maltreatment and peer victimization factor for adolescents. In addition, the current findings provide evidence that peer victimization loads with child maltreatment for both parents and adolescents. This was the case even with the measurement of peer victimization being more detailed among adolescents. As well, along with the original household challenges, evidence supports expanding these ACEs to include parental gambling, foster care or CPO contact, poverty, and neighborhood safety.

All original and expanded ACEs were associated with an increased likelihood of self-rated mental and/or physical health. The majority of these relationship remained significant after adjusting for sociodemographic variables. These findings provide further support for the CFA results as all individual ACEs in the original and possible expanded list were associated with indicators of poor self-rated health.

The following study limitations should be considered in interpreting the findings. First, the adolescent sample appears to be similar to the general population of with regard to sex, ethnicity, and income [58]. However, due to the non-random methods for data collection, we have not referred to the sample as representative. Second, due to the sensitive nature of some questions, adolescents were not asked about the full range of child maltreatment experiences. This resulted in not having assessments of physical and sexual abuse for adolescents. Third, exposure to IPV was measured differently for parents (physical) and adolescents (verbal). Fourth, we did not have an indicator of neighbourhood safety for the parents since we only asked about the current neighborhood and not the neighborhoods they lived in as children. Fifth, neighbourhood safety was the adolescent’s perception of safety and not the experience of neighbourhood violence. Sixth, we assessed peer victimization among both parents and adolescents; however, we used different items to assess these experiences including a much more detailed assessment of peer victimization among adolescents compared to parents. Notably, the peer victimization loaded similarly for parents and adolescents despite the difference in measurement. Seventh, we included both contact with CPO for parents and adolescents as well as placement in foster care for adolescents only. Again, despite this difference in assessment, the factor loadings were overall consistent for parents and adolescents. Eighth, adolescents were asked about their family having difficulty paying rent or the mortgage on the house and difficulty paying for basic necessities like food or clothing. It is possible that the adolescent would not have a full understanding of the family financial situation. Ninth, the timeframes for assessing ACEs were different for parents (i.e., when growing up or before age 16 years) and adolescents with few exceptions. The reason for this was that some adolescents were younger than 16 years at the time of the data collection. For consistency, we used a more current or past 12-month time frame for adolescents for most ACEs. However, the difference in the timeframe of the ACEs for parents and adolescents should be noted. Finally, the data were cross-sectional in nature and the assessments of ACEs were retrospective. However, the time period for recall is less among the adolescents and previous research has indicated that retrospective recall of adversity is research is a valid method [59].

## Conclusions

Based on the findings from the current study, it is recommended that the ACEs list should be expanded from 10 ACEs to 16 ACEs in two categories as follows: 1) child maltreatment and peer victimization ACEs, including physical abuse, sexual abuse, emotional abuse, emotional neglect, physical neglect, exposure IPV, spanking, and peer victimization, and 2) household challenges, including household substance use, household mental health problems, household gambling problems, parental separation or divorce, parental problems with police, foster care placement or CPO contact, poverty, and neighborhood safety (see Table 4). Having an expanded list of ACEs may help to better understand adversity in childhood and how it impacts on poor outcomes across the lifespan. However, this is not to mean that all future ACEs studies need to include all 16 ACEs.

**Table 4:** New Recommended Adverse Childhood Experiences (ACEs) list

Child Maltreatment and Peer Victimization	Household Challenges
Physical abuse	Household substance use
Sexual abuse	Household mental health problems
Emotional abuse	Household gambling problems
Emotional neglect	Parental separation or divorce
Physical neglect	Parental trouble with police
Exposure to intimate partner violence	Child protective organization contact or foster care placement
Spanking	Poverty
Peer victimization	Neighborhood safety

Notably, this study is not evidence for the validation of an ACEs tool. Rather, it provides evidence for the conceptualization of ACEs, how ACEs empirically group together, and how ACEs are related to poor mental and physical health outcomes. In fact, there has been very little innovation or development in how ACEs are measured and assessed over time. Further research in this area is encouraged to determine if these findings can be replicated using other data and if other ACEs should be added. Notably, the current findings show that all individual ACEs are related to poor mental and/or physical health indicators, but the effect sizes differ. The differing effect sizes may indicate that not all ACEs are equal with regard to associations with poor outcomes. This would be an argument against using ACEs scores or counts and rather highlights the importance the specific adverse experiences. Consistent with past research, it is likely the case that an increasing number of ACEs will show a dose-response trend with a health outcome with more ACEs experienced corresponding to greater likelihood of poor outcomes [7, 12, 60]. However, very little information beyond this can be generated. Since the current data show that individual ACEs may have larger or smaller effect sizes depending on the outcome, placing a focus on individual ACEs or co-occurrence of specific ACEs should be encouraged more so than the number of ACEs. It is important to continue to encourage innovative work that is empirically driven to replicate and extend knowledge in the ACEs field with the goal of furthering prevention efforts.

## Data Availability

Data are not publicly available. The WE Study data were not anonymous. Due to sensitive nature of the data and privacy and confidentially guidelines, the data must be housed in a secured lab and cannot be made publicly available.

## Abbreviations

ACEs: Adverse childhood experiences
CPO: Child protective organization
CFA: Confirmatory factor analysis
CFI: Comparative Fit Index
CI: Confidence interval
IPV: Intimate partner violence
OR: Odds ratio
RMSEA: Root Mean Square Error of Approximation
TLI: Tucker-Lewis Index
UVI: Unit variance identification
SRMR: Standardized Root Mean Square Residual
WE Study: The Well-Being and Experiences Study
WLSMV: Weighted least squares
